# A new operation technique for uterine prolapse: Vaginally-assisted laparoscopic sacrohysteropexy

**DOI:** 10.4274/tjod.96641

**Published:** 2017-09-30

**Authors:** İlhan Sanverdi, Çetin Kılıççı, Mesut Polat, Enis Özkaya, Sami Gökhan Kılıç, Merve Dizdar, Ateş Karateke

**Affiliations:** 1 University of Health Sciences, Zeynep Kamil Women and Children’s Health Training and Research Hospital, Clinic of Obstetrics and Gynecology, İstanbul, Turkey; 2 The University of Texas Medical Branch, Department of Obstetrics and Gynecology, Division of Female Pelvic Medicine and Reconstructive Surgery/Urogynecology, Texas, USA; 3 University of Health Sciences, Ümraniye Training and Research Hospital, Clinic of Obstetrics and Gynecology, İstanbul, Turkey

**Keywords:** Sacrohysteropexy, laparoscopy, vaginally-assisted laparoscopic sacrohysteropexy

## Abstract

**Objective:**

To describe the new surgical technique and report the safety and feasibility of vaginally-assisted laparoscopic sacrohysteropexy (VALSH).

**Materials and Methods:**

Thirty-three women with stage 3 or more uterine prolapse underwent VALSH operation. Patients were followed up for 12 months for mesh-related complications and improvements of symptoms. The operation had three sections; 1^st^ laparoscopic, 2^nd^ vaginal, 3^rd^ laparoscopic.

**Results:**

The mean age, gravidity, and parity of the study population were 46.5 years (range, 25-68 years), 4.3 (1-9), and 2.9 (1-6), respectively. The mean duration of operation was 59.5 min (range, 20-120 min). There were significant differences between the pre- and post-operative values of pelvic organ prolapse quantification parameters, which were favorable in the latter evaluation (p<0.001); total vaginal length was preserved after surgery (p>0.05).

**Conclusion:**

VALSH is a safe and minimally-invasive procedure in uterovaginal prolapse, with favorable anatomic and functional outcomes at 12 months post-operatively.

## PRECIS:

Vaginally-assisted laparoscopic sacrohysteropexy is a safe, minimally-invasive procedure in uterovaginal prolapse. Twelve months postoperatively, this procedure showed favorable anatomic and functional results.

## INTRODUCTION

Uterovaginal prolapse is a frequently encountered condition especially among older women, which may lead to disability and poor quality of life^(1)^, and it is a major indication for gynecologic surgery. The lifetime risk for a woman to have uterine prolapse surgery is 11%^(1)^. Risk of developing this kind of disorder increases with advancing age^(2)^. Surgery should be performed to restore anatomy with minimal morbidity and the lowest risk of recurrence. Three different approaches have been introduced to repair the pelvic floor such as abdominal, vaginal, and laparoscopic techniques. Hysterectomy is still considered to be the standard procedure for correcting uterovaginal prolapse^(3)^; however, in the majority of cases, hysterectomy does not overcome abnormalities associated with weakened pelvic support structures such as uterosacral and cardinal ligaments^(4)^. Additionally, due to the belief that the uterus plays a role in sexual satisfaction, an increasing number of women avoid undergoing hysterectomy^(5)^. Hysterectomy was shown to be associated with increased morbidity, blood loss, operative time, and influence post-operative recovery time^(6)^. Sacrospinous hysteropexy has been proposed to be an alternative approach for uterine-preserving prolapse surgery^(7)^. Other management alternatives include transvaginal mesh kits^(8,9)^, abdominal sacrohysteropexy using mesh^(10)^, and laparoscopic uterine suspension using a sling^(11)^ or mesh^(12)^. A modified form of uterine-preserving prolapse surgery using a combined vaginal and laparoscopic approach was introduced and a series of 70 women was reported by Fayyad and Siozos^(13)^.

In this case series, we describe the safety, feasibility, and outcomes of a modified form of a combined vaginal and laparoscopic approach, vaginally-assisted laparoscopic sacrohysteropexy (VALSH) for treating advanced uterovaginal prolapse.

## MATERIALS AND METHODS

This study is a descriptive prospective case series of 33 women with symptomatic stage 3 or 4 uterine prolapse on the pelvic organ prolapse-quantification (POP-Q) prolapse examination system^(14)^ who underwent VALSH between 2012 and 2015 in Zeynep Kamil Women and Children’s Health Training and Research Hospital. The procedure was approved by the Zeynep Kamil Women and Children’s Health Training and Research Hospital Local Ethics Committee (approval number: 2015/195) and informed consent forms were obtained from each patient. This new alternative of surgical intervention was offered to women with advanced uterine prolapse.

Inclusion criteria were as follows:

(1) Symptomatic uterine prolapse > stage 3; (2) symptoms of pelvic organ prolapse such as vaginal bulge and heaviness.

Women were asked to empty their bladder before examination. All examinations were performed while the women were in the lithotomy position. The women were asked to perform a maximum Valsalva maneuver before genital examination. Stages of uterine prolapse were determined according to the POP-Q classification. Preoperatively, all patients underwent POP-Q questionnaires to assess prolapsed-related symptoms^(15)^. After determining the surgical indication and following preoperative preparations, the procedure was performed under general anesthesia with the patient initially in the lithotomy position. Thirty-three women with stage 3 or more uterine prolapse underwent VALSH operations. Patients were followed up over a 12-month period after surgery for mesh-related complications and improvements of symptoms.

The operation consisted of three sections; 1^st^ laparoscopy, 2^nd^ vaginal, 3^rd^ laparoscopy. Laparoscopic ports were placed; one 10-mm port for umbilical and two or three 5-mm ports for lateral or suprapubic sites based upon conditions in order to achieve optimal surgical site exposure. The peritoneum over the sacral promontory was incised. A small 5-cm tunnel was made underneath the peritoneum from the sacral promontory downward to the cervix. Then, a lightweight type of mesh was placed onto the promontorium surface.

**Figure 1:**
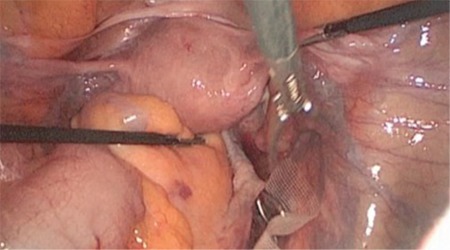
Laparoscopic view of curved ring forceps put into the retroperitoneal space via incision performed on the posterior wall of cervix

**Figure 2:**
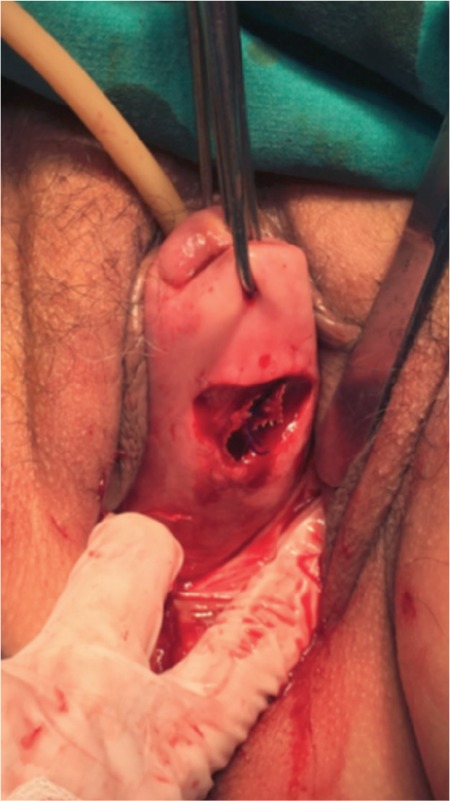
Cervical incision performed on the posterior wall where curved ring forceps are introduced and later mesh is fixed by 2-0 non-absorbable sutures x4

Second, the vaginal part of the surgery was performed. A semicircular incision was made at the posterior cervicovaginal junction. Curved ring forceps were placed into the retroperitoneal area via a tunnel established through blunt dissection from the vaginal part towards the promontory. At the same time, the direction of the instrument was visualized via laparoscopy (Figure 1). When the tip of the ring forceps was observed through the incision in the promontorium, the mesh was grasped and pulled downward within the tunnel with the aid of an instrument. The mesh was then fixed onto the posterior face of the uterine cervix using 3-4 non-absorbable sutures via the vaginal route (Figure 2). The vaginal incision was closed by absorbable sutures.

Finally, the uterus was pushed up to the maximum level using a Rubin’s cannula to obtain the required uterine suspension and mesh was tacked/sutured to the anterior longitudinal ligament at the sacral promontory and the peritoneal membrane over the promontory was sutured via the laparoscopic approach. A transobturator tape insertion procedure was applied in patients with stress urinary incontinence diagnosed before the operation through appropriate urogynecologic tests. A post-operative examination was performed in the lithotomy position using the POP-Q system. Mesh complications including mesh exposure were noted.

### Statistical Analysis

Data were analyzed using SPSS version 15.0 for Windows (SPSS Inc., Chicago, Illinois). The pre- and post-operative data were compared using the Wilcoxon t-rank test, and p values of <0.05 were considered significant.

## RESULTS

The mean age, gravidity, and parity of the study population were 46.5 years (range, 25-68 years), 4.3 (1-9), and 2.9 (1-6), respectively (Table 1). The mean operation time was 59.5 min (range, 20-120 min). Pre- and post-operative symptoms are shown in Table 2. The comparison of pre- and post-operative hemoglobin levels with the parameters of POP-Q values are summarized in Table 3. The rates of pre-operative prolapse stages were 4Ba (n=12, 36.3%), stage 4C (n=10, 30.3%), stage 3 Ba (n=3, 9.1%), stage 3C (n=3, 9.1%), stage 4 Bp (n=2, 6.1%), stage 4D (n=2, 6.1%), and stage 3 Bp (n=1, 3%). There were significant differences between the pre- and post-operative values of POP-Q parameters, which were favorable in the latter evaluation (p<0.001); total vaginal length was preserved after surgery (p>0.05). Among 33 women, stress urinary incontinence was determined in 12 patients by prolapse reduction testing (36.4%) managed by combined VALSH and transobturator tape insertion procedures. No perioperative complications were observed. There were five patients with cervical elongation concomitant with the uterine prolapse. At 12 months after surgery, all 33 patients reported cure of their prolapse symptoms with a subjective cure rate of 100%. None of the patients developed de novo urgency, infection or mesh erosion following surgery during the follow-up period. On assessing the patients’ global impression of improvement, all 33 women reported feeling either “very much better” or “much better.” No recurrence or mesh complication was observed after 12 months’ follow-up.

## DISCUSSION

In this case series, we wanted to show the feasibility of a new technique of VALSH. Data from our series show this technique to be feasible, safe, and easy to perform. It has several advantages over conventional techniques and recently introduced new vaginal-assisted laparoscopic approaches. There is still no consensus on the optimal management of advanced uterine prolapse. Vaginal hysterectomy has been proposed to be a standard procedure in these cases. In order to avoid removing a healthy organ and increasing morbidity and mortality with the hysterectomy procedure and also preserve fertility, uterine-preserving approaches were introduced a few decades ago^(10,11,12)^.

With the recent advances in endoscopic surgery, some laparoscopic uterine suspension techniques have been described^(16,17,18)^. According to the accumulated data, independent from the removal of the uterus, apical suspension is the required step for successful outcomes^(19)^. Recent studies showed some advantages of sacrohysteropexy including low recurrence rates, absence of mesh erosion, preserving an adequate vaginal length, and maintaining the proper physiological vaginal axis^(3)^. Additionally, laparoscopic hysteropexy was shown to be associated with better anatomic cure rates of higher than 90% in the majority of the studies. There was an improvement in symptoms, and cure rates of 73-100% among the patients. Reoperation rates and complication rates were generally low^(20)^.

Furthermore, endoscopic approaches have some well-defined advantages including quicker recovery and a reduction in adhesion formation^(12,13)^. Additionally, current laparoscopic techniques provide better and magnified visualization of the anatomy and better hemostasis resulting from intraperitoneal gas pressure^(13)^. In the literature, one of the techniques of laparoscopic ventrosuspension of uterus used the rectus sheath for this purpose. However, according to the reports from the literature, it had unsatisfactory results^(21)^. On the other hand, uterosacral plication and suture hysteropexy were reported to have 80% success rates^(19,22)^. Recently, total laparoscopic hysteropexy by mesh placement from the upper part of the cervix to the sacral promontory was developed^(12)^. In addition, a published cohort study on the long-term outcomes of laparoscopic versus vaginal mesh hysteropexy revealed high satisfaction rates for both procedures^(23)^. Most hysteropexy techniques have been shown to have high satisfaction and low reoperation rates. It was reported that the type of hysteropexy and possible graft configuration may impact reoperation rates for recurrent prolapse. Furthermore, authors claimed that vaginal mesh risks and the possibility of future hysterectomy with mesh-associated risks should also be considered^(24)^. Moreover, relative to conventional laparoscopic sacral hysteropexy, total laparoscopic hysterectomy with laparoscopic sacrocolpopexy procedures were shown to have similar anatomic results, excellent patient satisfaction, and improved quality of life scores^(25)^. Therefore, some modified forms of laparoscopic hysteropexy operations have been introduced.

In their series, Fayyad and Siozos^(13)^ aimed to report the results of a novel technique of hysteropexy using vaginal dissection and mesh placement and fixation of mesh to the sacral promontory via a laparoscopic view. It had some advantages as well as efficacy, especially in patients with cervical elongation and the extra-peritoneal attachment of the mesh to the cervix eliminated the risk of compromise of uterine blood flow. On the other hand, the theoretical disadvantage of this technique is that the insertion of the mesh vaginally can result in increased infection and mesh exposure rates^(13)^.

In their study, Fayyad and Siozos^(13)^ showed that their technique called “VALUES” was free from increased risk of vaginal shortening and narrowing. In addition, the procedure was shown to result in shorter hospital stay and quicker recovery^(13)^. Sacral colpopexy was reported to have a lower risk of recurrent prolapse on examination, redo surgery for prolapse, post-operative stress urinary incontinence, and dyspareunia than a variety of vaginal interventions. However, there is limited evidence to support the use of transvaginal mesh for apical vaginal prolapse repair. There is no consensus on the best access routes for sacral colpopexy. Moreover, there is no clear conclusion on the comparison between uterine-preserving surgery and vaginal hysterectomy for uterine prolapse^(26)^. Recent studies indicated the necessity of randomized controlled studies to show the benefits of laparoscopic sacrocervicopexy with or without supracervical hysterectomy in terms of surgical outcomes and reduced risk of mesh erosion compared with sacrocolpopexy and concomitant total hysterectomy^(25,27)^. Pelvic organ prolapses and stress urinary incontinence were reported to coexist in 80% of patients with pelvic floor dysfunction^(28)^. We detected coexistence of pelvic organ prolapses and stress urinary incontinence in 36.4% of cases, which was managed through transobturator tape insertion.

The most difficult part of the procedure during conventional sacrohysteropexy is to dissect the peritoneum down to the cervix. Hemorrhage during this dissection further deteriorates tissue exposure. Furthermore, this partially blinded dissection increases the risk of hypogastric nerve plexus injury. The cervical region in which the mesh is inserted is very close to the rectum, which further makes the procedure difficult. Also, it is difficult to fix the mesh low enough to the cervix in patients with cervical elongation, which results in unsatisfactory results.

### Study Limitations

This study has some limitations, one of them is small sample size and study needs longer follow-up duration.

## CONCLUSION

VALSH is a safe, minimally-invasive procedure in uterovaginal prolapse that preserves the uterus, enables future normal vaginal delivery, and has shown favorable anatomic and functional outcomes at 12 months follow-up including zero recurrence rates.

## Figures and Tables

**Table 1:**
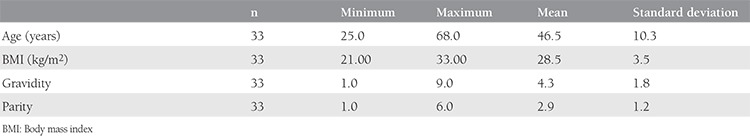
Summary of some demographic features of the study population

**Table 2:**
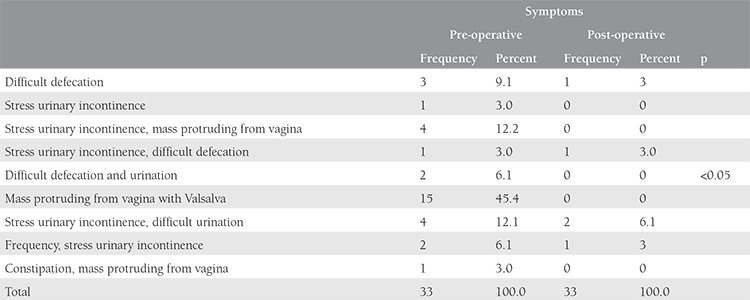
Summary of pre- and post-operative urogynecologic symptoms of the whole study population

**Table 3:**
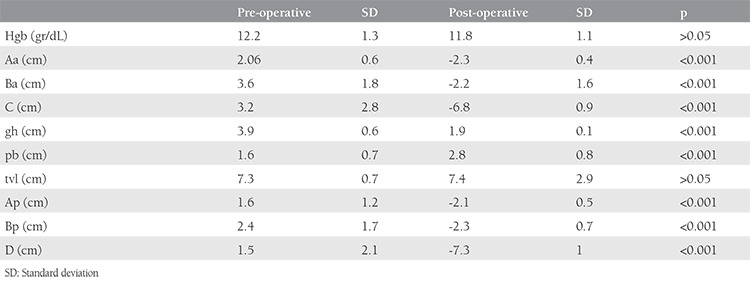
Comparison summary of pre- and post-operative pelvic organ prolapse-quantification classification parameters
